# Work hours and turnover intention among hospital physicians in Taiwan: does income matter?

**DOI:** 10.1186/s12913-016-1916-2

**Published:** 2016-11-21

**Authors:** Yu-Hsuan Tsai, Nicole Huang, Li-Yin Chien, Jen-Huai Chiang, Shu-Ti Chiou

**Affiliations:** 1 International Health Program, School of Medicine, National Yang-Ming University, Taipei, Taiwan; 2 Kaohsiung Veterans General Hospital, Kaohsiung, Taiwan; 3 Institute of Hospital and Health Care Administration, National Yang-Ming University, Taipei, Taiwan; 4 Institute of Community Health Care, National Yang-Ming University, Taipei, Taiwan; 5 Research & Development, Health Promotion Administration, Ministry of Health and Welfare, Taipei, Taiwan; 6 Health Promotion Administration, Ministry of Health & Welfare, Taipei, Taiwan; 7 School of Medicine, National Yang-Ming University, Taipei, Taiwan; 8 Taoyuan General Hospital, Ministry of Health & Welfare, 1492, Jhongshan Rd., Taoyuan District, Taoyuan City, 33004 Taiwan

**Keywords:** Hospital physicians, Turnover intention, Work hours, Pay satisfaction

## Abstract

**Background:**

Physician shortage has become an urgent and critical challenge to many countries. According to the workforce dynamic model, long work hours may be one major pressure point to the attrition of physicians. Financial incentive is a common tool to human power retention. Therefore, this large-scale physician study investigated how pay satisfaction may influence the relationship between work hours and hospital physician’s turnover intention.

**Methods:**

Data were obtained from a nationwide survey of full-time hospital staff members working at 100 hospitals in Taiwan. The analysis sample comprised 2423 full-time physicians. Dependent variable was degree of the physicians’ turnover intention to leave the current hospital. The pay satisfaction was assessed by physicians themselves. We employed ordinal logistic regression models to analyze the association between the number of work hours and turnover intention. To consider the cluster effect of hospitals, we used the “gllamm” command in the statistical software package Stata Version 12.1.

**Results:**

The results show that 351 (14.5%) of surveyed physicians reported strong intention to leave current hospital. The average work hours per week among hospital physicians was 59.8 h. As expected, work hours exhibited an independent relationship with turnover intention. More importantly, pay satisfaction could not effectively moderate the positive relationship between work hours and intentions to leave current hospital.

**Conclusions:**

The findings show that overtime work is prevalent among hospital physicians in Taiwan. Both the Taiwanese government and hospitals must take action to address the emerging problem of physician high turnover rate. Furthermore, hospitals should not consider relying solely on financial incentives to solve the problem. This study encouraged tackling work hour problem, which would lead to the possibility of solving high turnover intention among hospital physicians in Taiwan.

**Electronic supplementary material:**

The online version of this article (doi:10.1186/s12913-016-1916-2) contains supplementary material, which is available to authorized users.

## Background

Physician shortages are an emerging international concern [1–4]. More critically, there is no immediate solution to this problem. The workforce dynamic model, proposed by Sklar, suggests three phase of physician workforce dynamic and their various pressure points to which interventions can target [4]. Specifically, the attrition and outflow of physician threatens the functioning of health care systems. High physician turnover can lead to an inadequate supply of health services, thereby compromising people’s access to health care and endangering the quality of patient care and safety [5–7]. In addition, the adverse consequences of physician turnover may include financial loss, low patient satisfaction, and poor organizational practices [8].

Rapid changes in health care environments and increasing health care costs have led to great pressures on physicians. Hospital physicians may be particularly susceptible to these factors because of the nature and environment of their work. Compared with general practitioners, hospital physicians typically treat more patients with critical conditions, and they have a higher workload and lower job control. Furthermore, previous studies have indicated that psychosocial job stressors, high levels of distress, frequent sleep problems, heavy workloads, job dissatisfaction, and poor workplace relationships are plausible factors associated with high physician turnover [9–11]. Many of these work-related stress, distress, and dissatisfaction may be resulted partly from hospital physicians’ long work hours [12, 13].

The situation may be worse in many newly developed countries. Several studies have indicated that the average work hours among physicians in Taiwan are greater than those of physicians in other developed countries [14, 15]. Although some studies are available in Asia or in Taiwan, these studies suffer from the methodological issues such as low response rates, poor generalizability, and a lack of detailed analyses on the relationship between work hours and turnover intention. Because physicians play a crucial role in ensuring a well-functioning health care system, many countries (e.g., France, Canada, and the United States) have attempted to restrict the maximum work hours of resident physicians [16]. However, in newly developed countries, understanding of these problems remains limited and few efforts have been made to regulate the work hours of hospital physicians, especially in attending physicians.

From a management perspective, a possible and intuitive approach to employee retention is to increase income. The National Health Insurance program in Taiwan is famous for low expenditure, high medical service usage, high coverage rate, and acceptable medical quality [17]. Low expenditure with high usage rate, combined with ageing problem and increased cost due to technology advancement, undoubtedly put great pressure on hospital’s financial balance [18]. As a result, health professionals’ income is always the target of controlling cost, including physicians. Recently in Taiwan, malpractice lawsuit, excessive workload, and unfair income are three most frequently mentioned causes of the physician shortage in hospitals. Whether elevating the pay satisfaction of physician is a useful approach to decrease the impact of long work hours on turnover rate is a critical question for hospital managers and health policy makers.

## Methods

### Design and participants

#### Data source and study sample

In this cross-sectional study, we adopted the nationwide hospital survey *Needs Assessment Survey on Physical and Mental Health and Occupational Safety for Full-time Staff in Healthcare Workplace* (Additional file 1) which was conducted by Bureau of Health Promotion in 2011. The structured questionnaire survey was developed to assess the health, health-related behaviors, and work conditions of hospital staffs in Taiwan. Work hours and turnover intention are two main dimensions assessed in this survey. Previous research has shown that the questionnaire has exhibited acceptable validity and introduced its sampling method clearly (Additional file 1). Among the 127 selected hospitals, 100 (78.7%) agreed to participate in this survey, and all full-time staff members at these hospitals were requested to participate. The questionnaire was anonymous, and a return envelope was provided with each questionnaire. Hospital staffs were requested to return the completed questionnaires in a sealed envelope to collecting sites at the hospitals. We distributed 98,817 questionnaires from May 2011 to July 2011, and 70,622 (71.5%) validated questionnaires were returned. Among those who returned their questionnaires, 4538 respondents reported that they were physicians. After we excluded responses with incomplete information (678 people) and those below 35 years old (1437 people), the final sample in our study comprised responses from 2423 attending physicians. For those below 35 years old, they are very likely still under trainee. After accomplishing trainee, it is reasonable to change to other hospitals to pursue their new medical lives. To avoid overestimating the meaning of intention to leave current hospital as the willing of turnover, we only analyzed the physician group above 35 years old. Furthermore, following previous studies, we chose to focus on physicians’ intention to withdraw, instead of actual turnover, as the dependent variable for several considerations [11, 19]. First, the cross-sectional nature of the survey prevented the measurement of physicians’ actual turnover from practice. Second, previous studies have shown turnover intention is a reliable predictor of actual turnover [20, 21]. The study protocol was approved by the institutional review board at the Bureau of Health Promotion prior to distributing the survey (BHP investigation number 0990800708).

### Measurements

#### Dependent variable

Turnover intention was assessed using one question (“What is the likelihood that you will leave your current hospital?”. The responses were measured using a 5-point Likert scale ranging from 1 (*none*) to 5 (*very strong*). We classified turnover intention as *mild* (i.e., physicians who answered *none* or *mild*), *moderate* (i.e., physicians who answered *moderate*), or *strong* (physicians who answered *strong* or *very strong*).

#### Independent variable and moderating variable

The main independent variable was the number of work hours, which was measured using the survey item “Please recall how many hours you worked in the last week”. According to Taiwan Labor Standards Act, the normal weekly work hours shall not exceed 48 h, and the overtime weekly work hours shall not exceed 60 h [22]. Physicians in Taiwan are not considered laborers; thus, the Labor Standards Act does not apply to this profession. No legislation exists to regulate hospital physicians’ work hours, so the proposed reference of an 88-h maximum for residents’ work hours per week by the American ACGME (The Accreditation Council for Graduate Medical Education) was also adopted as one cut point [23]. Based on those cut points, we categorized the sample into five groups (< 49, 49–59, 60–88, and >  88 h) to further evaluate the impact of work hours on turnover intention.

The moderating variable considered in this study was pay satisfaction, which was assessed by one question (“Do you think it is reasonable for your current work pay?”. The responses were measured using a 5-point Likert scale ranging from 1 (very unreasonable) to 5 (very reasonable). We classified pay satisfaction as bad (i.e., physicians who answered very unreasonable or little unreasonable), moderate (i.e., physicians who answered moderate), or good (physicians who answered not bad or very reasonable).

#### Control variables

In the analyses, we also included sociodemographic variables (age, gender, and marital status) and work characteristics variables (seniority at current hospital, clinical setting, supervisor position, accredited hospital level, hospital ownership, and health promoting hospital (HPH) status). Furthermore, we subsequently included health status and job satisfaction as control variables to adjust their impact on the relationship between work hours and turnover intention. Self-rated health status was separately categorized into three groups (good, moderate, and bad), and job satisfaction was divided into two groups (good and bad).

### Statistical analyses

We employed ordinal logistic regression models to analyze the association between the number of work hours and turnover intention. This method is suitable for dependent variables with multiple ordered response categories, and we verified the appropriateness of using this model, which means the relationship between any two pairs of outcome groups is statistically the same. To consider the cluster effect of hospitals, we used the “gllamm” command in the statistical software package Stata Version 12.1(StataCorp, 4905 Lakeway Drive College Station, Texas 77845-4512 USA), which can estimate generalized linear latent and mixed models. First, multivariable regression was applied to determine the relationship between work hours and turnover intention by adjusting the sociodemographic and work characteristics variables (Model 1). Next, we analyzed the association between work hours and turnover intention by incorporating health status, pay satisfaction, and job satisfaction into the models (Model 2). Finally, to evaluate whether pay satisfaction moderated the relationship between work hours and turnover intention, we conducted stratification analyses based on the level of pay satisfaction.

## Results

Table 1 lists the distribution of the physicians’ characteristics and weekly work hours, showing that 351 (14.5%) of the surveyed physicians reported strong intention to leave their current hospital. There were up to 727 (30.0%) physicians rating pay satisfaction as bad. The average weekly work hours among Taiwan hospital physicians was 59.8 (19.9). Regarding the categories for the number of work hours per week, 1102 (45.5%) of the physicians worked more than 60 h, and 207 (8.5%) physicians worked more than 88 h. Longer work hours were associated with a stronger intention to leave a hospital. Additionally, the number of work hours decreased as age increased. On average, women worked 6 h less per week than their male counterparts. Those who were working in surgery or delivery rooms, emergency or intensive care unit reported a relatively much higher number of weekly work hours as 66.0 and 65.0, respectively. Physicians who were employed at medical centers reported working much longer hours than their counterparts in regional or district hospitals as 64.4 h per week. In addition, as average weekly work hours increased, self-rated health status, pay satisfaction, and job satisfaction deteriorated significantly. Table 2 shows that longer work hours and lower pay satisfaction were both related to higher turnover intention to leave current hospital, and the impact was statistically significant (*P* value < 0.001) and substantial.

**Table 1:** Physician Characteristics and Work Hours Distribution

	All doctors (*N* = 2423)		Work hours (*N* = 2423)		
	*N*	%	Mean	SD	*P* value
Dependent Variables					
Leave hospital					*P* < 0.001
mild	1328	54.8	58.3	19.3	
moderate	744	30.7	60.1	19.3	
strong	351	14.5	64.9	22.3	
Independent Variables					
Work hours			59.8	19.9	
< =48	718	29.6			
49–59	603	24.9			
60–88	895	36.9			
> =89	207	8.5			
Age					*P* < 0.001
35–49	1702	70.2	60.9	20.3	
> =50	721	29.8	57.2	18.5	
Gender					*P* < 0.001
male	2026	83.6	60.8	19.8	
female	397	16.4	54.7	19.4	
Marital status					*P* = 0.058
unmarried	213	8.8	58.9	19.8	
married	2136	88.2	59.7	19.5	
Others	74	3.1	65.1	28.3	
Seniority at current hospital					*P* = 0.247
< =1 year	210	8.7	59.4	22.0	
2–5 years	533	22.0	61.1	20.4	
> 5 years	1680	69.3	59.5	19.4	
Clinical setting (1)					*P* < 0.001
others	1624	67.0	56.8	17.8	
surgery or delivery room	799	33.0	66.0	22.3	
Clinical setting (2)					*P* < 0.001
others	1652	68.2	57.4	18.6	
emergency or ICU	771	31.8	65.0	21.5	
Supervisor					*P* = 0.231
supervisor	879	36.3	60.5	19.1	
on-Supervisor	1544	63.7	59.5	20.3	
Accredited hospital level					*P* < 0.001
district hospital	159	6.6	54.4	16.3	
regional hospital	1711	70.6	58.8	19.5	
medical center	553	22.8	64.4	21.2	
Hospital ownership					*P* = 0.008
public hospital	901	37.2	58.4	18.7	
private hospital	1522	62.8	60.6	20.5	
Health Promoting Hospital (HPH) status					*P* = 0.396
Non-HPH	1057	43.6	60.4	20.0	
HPH	805	33.2	59.4	19.6	
Exemplary HPH	561	23.2	59.2	20.0	
Health status					*P* < 0.001
good	510	21.1	57.6	19.0	
moderate	1471	60.7	59.8	19.7	
bad	442	18.2	62.6	21.0	
Pay satisfaction					*P* < 0.001
good	452	18.7	56.1	16.8	
moderate	1244	51.3	58.8	19.1	
bad	727	30.0	63.8	22.2	
Job satisfaction					*P* < 0.001
good	1804	74.5	58.3	18.6	
bad	619	25.6	64.3	22.5

**Table 2:** Crude ratio of intention to leave hospital with work hours and pay satisfaction

	Intention to leave current hospital (*N* = 2423)				
	Mild	Moderate	Strong	Total	*P* value
Work hours					*P* < 0.001
<=48					
*N*	435	207	76	718	
%	60.6	28.8	10.6	100.0	
49–59					
*N*	331	183	89	603	
%	54.9	30.4	14.8	100.0	
60–88					
*N*	473	286	136	895	
%	52.9	32.0	15.2	100.0	
> = 89					
*N*	89	68	50	207	
%	43.0	32.9	24.2	100.0	
Pay satisfaction					*P* < 0.001
Good					
*N*	335	89	28	452	
%	74.1	19.7	6.2	100.0	
Moderate					
*N*	737	409	98	1244	
%	59.2	32.9	7.9	100.0	
Bad					
*N*	256	246	225	727	
%	35.2	33.8	31.0	100.0	

Table 3 shows the association between work hours and intention to leave the current hospital. After we controlled for other variables, work hours exhibited a independent relationship with turnover intention. After adjusting for socio-demographic variables and work characteristics (Model 1), our results indicated that in comparison with physicians who worked less than 49 h per week, those who worked 60–88 h (OR, 1.40; 95% CI, 1.14–1.72) and more than 88 h (OR, 2.09; 95% CI, 1.52–2.87) had significantly stronger intentions to leave their current hospital. After we added health status, pay satisfaction, and job satisfaction into the model (Model 2), the positive relationship between work hours and intention to leave their current hospital persisted, although the magnitude of the effects reduced moderately for both the 60–88 h group (OR, 1.22; 95% CI, 0.98–1.51) and the more than 88 h group (OR, 1.53; 95% CI, 1.10–2.13). Furthermore, in multivariate analysis, young physicians, moderate seniority at current hospital (2–5 years), poor self-perceived health status, pay dissatisfaction, and job dissatisfaction were significant predictors of strong intention to leave current hospital.

**Table 3:** Work hours and Intentions to leave current hospital

	Intentions to leave current hospital (N = 2423)					
	Simple Ordinal Logistic Regression		Model 1		Model 2	
	OR	95% C.I.	OR	95% C.I.	OR	95% C.I.
Work hours (REF = <=48)						
49–59	1.28*	1.03–1.59	1.31*	1.05–1.63	1.21	0.96–1.52
60–88	1.40**	1.15–1.71	1.40**	1.14–1.72	1.22	0.98–1.51
>–89	2.29***	1.69–3.11	2.09***	1.52–2.87	1.53*	1.10–2.13
Age (REF= > =50)						
35–49	2.02***	1.68–2.42	1.85***	1.53–2.23	1.73***	1.42–2.11
Gender (REF = male)						
Female	1.01	0.82–1.25	0.98	0.78–1.22	1.08	0.85–1.36
Marital status (REF = unmarried)						
Married	0.75*	0.57–0.98	0.86	0.65–1.14	1.19	0.88–1.60
Others	1.11	0.66–1.86	1.30	0.77–2.20	1.42	0.82–2.46
Seniority at current hospital (REF = <=1 year)						
2-5 years	1.68**	1.22–2.32	1.65**	1.19–2.29	1.49*	1.06–2.10
> 5 years	1.30	0.97–1.74	1.51**	1.11–2.05	1.36	0.99–1.87
Clinical setting (1) (REF = others)						
Surgery or delivery room	1.05	0.88–1.24	0.94	0.79–1.13	0.91	0.75–1.09
Clinical setting (2) (REF = others)						
Emergency or ICU	1.36***	1.15–1.61	1.16	0.97–1.39	0.97	0.81–1.17
Supervisor (REF = supervisor)						
Non-supervisor	1.45***	1.23–1.71	1.31**	1.10–1.57	1.09	0.91–1.31
Accredited hospital level (REF = district hospital)						
Regional hospital	1.07	0.72–1.57	1.03	0.69–1.52	1.12	0.76–1.66
Medical center	0.97	0.61–1.53	0.95	0.59–1.52	0.93	0.58–1.47
Hospital ownership (REF = public)						
Private hospital	0.80*	0.65–0.99	0.82	0.66–1.03	0.81	0.65–1.01
HPH status (REF = Non-HPH)						
HPH	1.19	0.94–1.51	1.18	0.93–1.51	1.03	0.81–1.30
Exemplary HPH	0.95	0.71–1.26	0.96	0.72–1.28	0.83	0.63–1.09
Health status (REF = good)						
Moderate	1.70***	1.37–2.09			1.24	0.99–1.55
Bad	3.15***	2.43–4.08			1.59**	1.21–2.10
Pay (REF = good)						
Moderate	1.87 ***	1.47–2.38			1.51**	1.18–1.94
Bad	6.33***	4.88–8.21			2.61***	1.96–3.48
Job satisfaction (REF = good)						
Bad	7.34***	6.05–8.91			4.69***	3.76–5.85

HPH: Health Promoting Hospital

*P < 0.05 **P < 0.01***P < 0.001

To determine whether pay satisfaction moderated the association between work hours and turnover intention, we conducted stratification analyses (Table 4). The result indicated that among those who perceive their income as good or bad, the relationship between work hours and turnover intention disappeared. However, for those who stated moderate pay satisfaction, which comprised the majority of physicians (51.3%), work hours remained strong independent relationship with turnover intention. Those who worked 60–88 h (OR, 1.57; 95% CI, 1.15–2.13) and more than 88 h (OR, 2.00; 95% CI, 1.21–3.32) had significantly stronger intentions to leave their current hospital comparing with physicians who worked less than 49 h per week. However, the overall interaction P value of pay satisfaction and work hours did not reach the level of statistical significance (*P* value = 0.447).

**Table 4:** Pay satisfaction stratification analysis

				Intention to leave current hospital (*N* = 2423)			
	*N*		Mild	Moderate	Strong	Model 2	
	Total (%)		*N* (%)			OR	95% C.I.
Good pay satisfaction	452 (100)						
Work hours							
< =48	161 (35.6)	100%	120 (74.5)	31 (19.3)	10 (6.2)	1	
49–59	115 (25.4)	100%	87 (75.7)	22 (19.1)	6 (5.2)	0.85	0.46–1.56
60–88	156 (34.5)	100%	115 (73.7)	32 (20.5)	9 (5.8)	1.05	0.59–1.86
> =89	20 (4.4)	100%	13 (65.0)	4 (20.0)	3 (15.0)	1.02	0.33–3.13
Moderate pay satisfaction	1244 (100)						
Work hours							
< =48	381 (30.6)	100%	250 (65.6)	115 (30.2)	16 (4.2)	1	
49–59	331 (26.6)	100%	190 (57.4)	108 (32.6)	33 (10.0)	1.52*	1.10–2.11
60–88	440 (35.4)	100%	249 (56.6)	154 (35.0)	37 (8.4)	1.57**	1.15–2.13
> =89	92 (7.4)	100%	48 (52.2)	32 (34.8)	12 (13.0)	2.00**	1.21–3.32
Bad pay satisfaction	727 (100)						
Work hours							
< =48	176 (24.2)	100%	65 (36.9)	61 (34.7)	50 (28.4)	1	
49–59	157 (21.6)	100%	54 (34.4)	53 (33.8)	50 (31.9)	1.08	0.71–1.64
60–88	299 (41.1)	100%	109 (36.5)	100 (33.4)	90 (30.1)	0.93	0.64–1.35
> =89	95 (13.1)	100%	28 (29.5)	32 (33.7)	35 (36.8)	1.14	0.68–1.91

This table has adjusted the impact of age, gender, marital status, seniority at current hospital, clinical setting, supervisor, hospital level, hospital ownership, health promoting hospital status, health status, and job satisfaction. (2) The interaction P value is 0.447, which was far away from achieving statistically significant level

*P < 0.05 **P < 0.01

## Discussion

This is the first article to directly analyze the effect of work hours on turnover intention, and also put pay satisfaction into consideration to estimate the possible moderating effect. There are three interesting findings. First, the average work hours among hospital physicians who are above 35 years old in Taiwan was 59.8 (19.9) hours per week, which was considerably higher than that of physicians in United States (49.6 h per week for nonresident physicians) [24]. More importantly, 214 (8.6%) of the surveyed physicians worked more than 88 h per week. Second, a clear independent relationship was observed between work hours and turnover intention. These findings not only raise serious concerns regarding the health and wellbeing of physicians but also indicate that overtime work may increase physicians’ intentions to leave their current practice; thus, adding unnecessary administrative cost and more critically, endangering the continuity of patient care. The physician shortage problem in the hospital sector may threaten the function of the health care system. Unlike other factors associated with physicians’ turnover intentions (e.g., work stress, job satisfaction, etc.), work hours is a tangible and actionable factor. By building a reasonable work hour regulation, we can improve the wellbeing of physicians and diminish the possibility of leaving current practice setting.

Third, we know that even though financial incentive plays an important role in retaining physicians, it still cannot effectively moderate the adverse effect of long work hours on turnover intention. Similar results have been proposed by previous studies. One cross-sectional study in Ghana revealed that the dimensions of motivation and job satisfaction significantly associated with turnover intention included career development, workload, management, organizational commitment, and burnout, but not remuneration [25]. Another article conducted in England to retain the general practitioner workforce showed that reduced intensity and volume of workload were more important than incentive payment [26]. A review article to survey incentives for retaining health workers also documented financial incentives alone would exert limited effort. Working conditions, supervision and management, and education and training opportunities were also important and needed to be addressed together [27]. As a result, hospital managers and government should not overlook the influential role of work hours. Money cannot solve everything, and there are still root causes which drive the turnover intention of physician, such as long work hours. Taking action on improving the overload of work can assist elevating the wellbeing of physicians, and resulting in mitigating turnover intention, which assure the well function of health system.

However, there were some limitations in this study. First, because this was a cross-sectional study, we were unable to ascertain the causal relationship between work hours and turnover intention, which could be addressed by future longitudinal studies. Second, because of data limitations, some relevant information, such as the physicians’ rank (intern, resident, fellow, or attending physician) or specialty were unavailable, thus preventing detailed analyses. However, we have tried to use the variables of age and clinical settings to offer more information. Third, because we were not assured the exact number of physicians who attained the questionnaire, it’s impossible to calculate the specific physicians’ response rate in this study. Forth, work hours were self-reported and based on recall, which may have resulted in the figures being over-reported or under-reported because of perceived differences related to *work*. If the reporting is non-differential, the effects of work hours may be stronger than we observed. Besides, some points require further discussion. Although we used weekly work hours as our primary independent variable, the concept of *work hours* should be refined to elucidate the turnover problem further. For example, the number of work hours is not necessarily representative of work intensity [28]. Other variables (e.g., job control and work schedule flexibility) may also affect the psychological health of physicians [29]. The stress feeling at work, the interruptions and other variables regarding work situation was incomplete, thus, hinder further understanding about the true root of work hour issue. Moreover, the intentions to retire, take a career break, or reduce clinical hours of work are important questions about physician workforce which were lack in the survey. Finally, although we sought to include as many as possible confounding variables in the analyses according to literature review, there may still be other factors that need to be explored further. For future studies, post-questionnaire focus groups or individual one-to-one interviews would be useful to get a more in-depth understanding of the factors responsible for employment unhappiness.

In order to implement the findings of this study, we may need to rethink of the medical system. If some physicians quit form the job due to long work hours, then those who stay in position will encounter more work hours, and thus lead to stronger intention of leaving their hospital. From the results of this article, we should break up the pernicious circle between long work hours and turnover intention. However, if cutting number of hours each physician does during a week is the proposed solution, then more physicians will presumably be needed to fulfill the same workload as now. Some plausible methods should be attempted to solve the possible workforce shortage problem. For instance, task shifting to replacement medical staff, such as enhancing the role and increasing the number of nurse practitioners or physician assistants may be suitable. Developing more effective information systems may help physicians to work more efficiently during their work hour. Strengthening the ability of primary care system and spreading the ideas of integrated medicine will also reduce the workload of hospital specialty physicians. Based on above method or other innovative solutions, we can reduce physician’s long work hours without necessarily increasing the total number of physicians.

## Conclusions

In this study, we conducted a large-scale survey of hospital physicians. The sample comprised 2423 physicians working at 100 hospitals in Taiwan, assuring good generalizability of the study. The medical centers and regional hospitals where physician shortages were the most serious in Taiwan were included in the sample. The number of total hospital physicians working in Taiwan in 2011 was 24,552, and this study included 9.9% of them to conduct analyses [30]. The sample size of 2423 physicians ensured high statistical power, not only in determining the significant effects of work hours on turnover intentions but also in examining whether pay satisfaction moderates this association. The findings show that overtime work is prevalent among hospital physicians in Taiwan. Both the Taiwanese government and hospitals must take action to address the emerging problem of physician high turnover rate. Regularly assessing physicians’ work conditions (e.g., work hours), and limiting excess work hours may be suitable policy tools. Furthermore, hospitals should not consider relying solely on financial incentives to solve the problem. There was a clear need to face the overload work hours of hospital physicians. Although it may be difficult and challenging, this study encouraged tackling work hour problem, which would lead to the possibility of solving high turnover intention among hospital physicians in Taiwan.

## Additional file

Additional file 1: A preliminary English version of the questionnaire “Needs Assessment Survey on Physical and Mental Health and Occupational Safety for Full-time Staff in Healthcare Workplace”. (PDF 312 kb)
