# Supplementary material for: Work hours and turnover intention among hospital physicians in Taiwan: does income matter?
Source: BMC Health Serv Res. 2016 Nov 21;16:667. doi: 10.1186/s12913-016-1916-2 (PMC5117625; doi:10.1186/s12913-016-1916-2)
Supplement: Additional file 1: — A preliminary English version of the questionnaire “Needs Assessment Survey on Physical and Mental Health and Occupational Safety for Full-time Staff in Healthcare Workplace”. (PDF 312 kb) [file 12913_2016_1916_MOESM1_ESM.pdf]

Needs Assessment Survey on Physical and Mental Health and Occupational Safety for Full-time Staff in Healthcare Workplace

■ **Personal Health Needs**

● **Healthy Lifestyle**

1. **Physical Activity**: Taking the amount equivalent to total brisk walking time for more than 30mins per day as a minimum, how many days do you achieve in last week?
   (1) 0 day (2) 1-2 days (3) 3-4 days (4) 5-6 days (5) 7 days
2. **Diet**: How many days do you consume fruits in last week?
   (1) 0 day (2) 1-2 days (3) 3-4 days (4) 5-6 days (5) 7 days
3. Taking 5 servings of **vegetables or fruits** as a target, how many days do you achieve in last week?
   (1) 0 day (2) 1-2 days (3) 3-4 days (4) 5-6 days (5) 7 days
4. Will you think of **calories or nutrition** when you eat or purchase foods?
   (1) Never (2) Seldom (3) Sometimes (4) Usually (5) Always
5. Will you look at **calories labeling** when you are buying food?
   (1) Never (2) Seldom (3) Sometimes (4) Usually (5) Always
6. Will you look at **nutrition panel** for items other than calories when you are buying food?
   (1) Never (2) Seldom (3) Sometimes (4) Usually (5) Always
7. Body height: __ __ __cm
8. Body weight: __ __ __kg
9. How do you see your body shape now?
   (1) Skinny (2) Thin (3) Moderate (4) Overweight (5) Obesity
10. **Body weight control**: Have you ever tried to restrain your diet or control body weight in the past year?
   (1) Never (2) Seldom (3) Sometimes (4) Usually (5) Always
11. **Sleep time**: How many hours did you sleep on average at night in the past year?
   (1) Less than 5 hours (2) Around 6 hours (3) Around 7 hours (4) More than 8 hours
12. **Smoking** behavior:
   (1) Never (2) Ever, but have quitted (3) Smoker now

12-1. Do you consider or prepare to quit smoking?
   (1) Never (2) Consider to quit (3) Ready to quit (4) Quitting now

13. **Drinking** behavior:
   (1) Never (2) Sometimes (3) Usually small amount (4) Usually large amount
14. **Betal nut chewing:**
   (1) Never (2) Seldom (3) Sometimes (4) Usually (5) Always

- **Stress and Work**

15. Overall, what's your **work stress** level **in past year**?
    (1) None (2) Mild (3) Moderate (4) Strong (5) Very strong
16. Please recall **how many hours you worked in the last week**: __ __ __ hours/week
17. Please identify the stress level from each of the following work stressor? **Please choose the most suitable level**.
    (1) None (2) Mild (3) Moderate (4) Strong (5) Very strong

Personal relationship
Patient
Own department's supervisor
Colleague
Other departments' working staff
Hospital director
External policy
Board of directors
National health insurance
Hospital accreditation
Health policy
Work characteristics
Workload and work time
Physical burden
Difficulty and complexity of work
Uncertainty of medical outcomes
Danger of work
Death and sorrow attack during work
Work conditions
Unfamiliarity and under-trained of work
Lack guidance and support from work
Lack mental support
Urgency
Performance requirement
Lack work safeguard
Inequity of work demand
Promotion

18. Do you have advanced **training opportunities** in current work?
    (1) None (2) Little (3) Moderate (4) Many (5) A lot of

19. Do you have **positive expectation** on your **future career development** in your current hospital?
    (1) None (2) Little (3) Moderate (4) Not bad (5) Yes, very high
20. Do you think it is reasonable for your current work **pay**?
    (1) Very unreasonable (2) Little unreasonable (3) Moderate (4) Not bad (5) Very reasonable
21. Do you feel a sense of **accomplishment** in your current work?
    (1) None (2) Little (3) Moderate (4) Not bad (5) Yes, very high
22. Are you **satisfied** with current work?
    (1) Very unsatisfied (2) Little unsatisfied (3) Moderate (4) Not bad (5) Very satisfied
23. Do you feel depressed or down **this week** (including today)?
    (1) None (2) Mild (3) Moderate (4) Strong (5) Very strong
24. What is the likelihood that you will **leave your current hospital**?
    (1) None (2) Mild (3) Moderate (4) Strong (5) Very strong
25. What is the likelihood that you will **change your occupation**?
    (1) None (2) Mild (3) Moderate (4) Strong (5) Very strong
26. Do you agree that **health promotion and disease prevention of hospital staff** should be considered as an important aspect of hospital:
    (1) Very disagree (2) Little disagree (3) No opinion (4) Agree (5) Very agree
27. Please choose "five health issues" that you want your hospital to promote most:
    (1) **Exercise**
    (2) **Healthy diet**
    (3) **Weight control**
    (4) **Smoking** cessation
    (5) **Alcohol** quitting
    (6) Stress coping and **mental** hygiene
    (7) **Regular health checkup** (eg. adult health check, cancer screening)
    (8) Improve **NCDs** control (eg. hypertension, diabetes, dyslipidemia)
    (9) Prevention and handle of **low back pain**
    (10) Prevention and handle of **needle injury**
    (11) **Radiation** protection
    (12) Prevention pollutants of **chemical drug**
    (13) **Noise** prevention
    (14) **Occupational safety and violence protection** of medical staff
    (15) Prevention of **falling down, sprain injury**
    (16) Others: for example ______________________

- **Health conditions**

28. **Overall**, what do you think about your **current health status**?
    (1) Very bad (2) Not good (3) Moderate (4) Good (5) Very good
29. **Non-communicable diseases (NCDs)**: Do you have the following disease (can choose more than one answer)?
    (0) None (1) Hypertension (2) Diabetes (3) Dyslipidemia (4) Viral hepatitis
    (5) Gout (6) Insomnia (7) Asthma (8) Fatty liver (9) Others:____________
30. The time from last **health checkup** (eg. Adult health checkup, labor/civil servant/self-paid health checkup) to present:
    (1) Never done (2) More than 6 years (3) 4~6 years (4) 1~3 years (5) Less than 1 year
31. The time from lastest **stool occult blood** examination to present:
    (1) Never done (2) More than 4 years (3) 2~4 years (4) Less than 2 year
32. The time from lastest pap smear examination to present:
    (1) More than 6 years (2) 4~6 years (3) 1~3 years (4) Less than 1 year
    (5) I am **female above 30 y/o**, never done
    (6) I am **female below 30 y/o**, never done
    (7) I am male
33. The time from lastest **mammography** examination to present:
    (1) Less than 2 years (2) 2~4 years (3) More than 4 years
    (4) I am **female above 40 y/o**, never done
    (5) I am **female below 40 y/o**, never done
    (6) I am male

- **Occupational safety & protection**

34. Do you have **low back pain** currently? (1) No (2) Yes
35. Do you ever **sprain or strain in hospital** in past year? (1) No (2) Yes
36. Do you ever **fall in hospital** in past year? (1) No (2) Yes
37. Do you ever hurt by **needle in hospital** in past year? (1) No (2) Yes, ______ times
38. Do you ever hurt by **blade or sharp tool (not including needle)** due to **work** in hospital in past year? (1) No (2) Yes, ______ times
39. Is there **noise disturbanc**e in your **workplace**?
    (1) None (2) Little annoying (3) Moderate (4) Annoying (5) Very annoying
40. Have you ever exposed to **radiation without protection** during work in past year?
    (1) None or unknown (2) 1-2 times (3) More than 3 times

41. Have you ever exposed to **anesthesia gas or chemotherapy drug** during work in past year?
    (1) None or unknown (2) 1-2 times (3) More than 3 times
42. Have you ever suffered from **physical violence** in hospital in past year?
    (1) None or unknown (2) 1-2 times (3) More than 3 times
43. Have you ever suffered from **threatening of personal safety** in hospital in past year?
    (1) None or unknown (2) 1-2 times (3) More than 3 times
44. Have you ever suffered from **verbal or sexual violence or invasion** in hospital in past year?
    (1) None or unknown (2) 1-2 times (3) More than 3 times
45. Have you ever hurt by or suffered from **other causes** related to work?
    (1) No (2) Yes (Please explain): ____________________________
46. Have you ever made **sick leave** in past year?
    (1) None (2) Yes, ____ days
47. Have you ever made leave due to **physical or mental discomfort** caused by **work** in past year?
    (1) None (2) Yes, ____ days

■ **Exercise environment**

1. What is the level of health promotion activity in your hospital in encouraging hospital staff to exercise?
   (1) None (2) Little (3) Some (4) Much (5) Very strong
2. How often did you attend a course or activity related to **exercise** in hospital in the past year?
   (1) None (2) Sometimes (several times) (3) Usually
3. Have you ever done **physical fitness test** in your hospital?
   (1) No (2) Yes
4. Are there ways to encourage hospital staff to **climb stairs more often** in your hospital? (eg. Encouraging logos in front of the elevator; Improve lightening or decorations of stairs; Arrange contest of climbing stairs)
   (1) None (2) Few (3) Some (4) Many (5) A lot of
5. Do you usually **climb stairs** in your hospital?
   (1) Never (2) Seldom (3) Sometimes (4) Usually (5) Everyday
6. Is there any **exercise or fitness equipment inside or outside hospital** for staff to use in your hospital? (eg. Treadmill, Sports bicycle, Court, Swimming pool, Wii, etc)
   (1) None (2) Little (3) Some (4) Much (5) A lot of

7. Have you **ever used fitness or exercise equipment in** your hospital (inside or outside hospital)?
   (1) Never (2) Less than 1 time/ month (3) More than 1 time/ month
   (4) 1-2 times/ week (5) More than 3 times/ week
8. Is there **any fitness club** in your hospital?
   (1) None (2) Few (3) Some (4) Many (5) A lot of
9. Have you ever **joined** a fitness club in your hospital?
   (1) Never (2) Less than 1 time/ month (3) More than 1 time/ month
   (4) 1-2 times/ week (5) More than 3 times/ week
10. Does your hospital promote **physical exercise**?
   (1) No (2) Only play the music (3) Promote at specific departments
   (4) Promote at most departments (5) Promote at every department
11. Have you **ever done** physical exercise in your hospital?
   (1) Never (2) Less than 1 time/ month (3) More than 1 time/ month
   (4) 1-2 times/ week (5) More than 3 times/ week
12. Do you think you have done sufficient level of exercises?
   (1) Very lack (2) Not enough (3) Moderate (4) Enough (5) Very enough
13. Overall, are you satisfied with **exercise environment** of your hospital?
   (1) Very unsatisfied (2) Little unsatisfied (3) Moderate (4) Not bad (5) Very satisfied
14. In the aspect of exercise environment, what do you want your **hospital to do more**? (You can choose multiple items)
   (1) None
   (2) Increase availability of fitness equipment
   (3) Promote and educate exercise related issues
   (4) Encourage the utilization of stairs
   (5) Hold working staff sports competition
   (6) Offer physical fitness test
   (7) Hold regular exercise-related activities or competition
   (8) Play health exercise music to promote exercise
   (9) Provide discount or incentives for exercise club participation or exercise facility usage
   (10) Others: for example ______________________

■ **Diet environment**

1. Is there any **promotion activity in encouraging healthy diet** for hospital staff in your hospital?
   (1) None (2) Little (3) Some (4) Much (5) Very strong

2. Have you ever attended a **lecture** or activity related to **nutrition, diet, or cooking** in your hospital in the past year?
   (1) None (2) Sometimes (several times) (3) Usually
3. Is it easy for you to buy **healthy diet** (low fat, low salt, high fiber) **in your hospital**?
   (1) Very difficult (2) Not easy (3) Moderate (4) Easy (5) Very easy
4. Is it easy for you to access to **healthy diet in communities** surrounding your hospital?
   (1) Very difficult (2) Not easy (3) Moderate (4) Easy (5) Very easy
5. Have you ever **attended activities or lectures** on **weight control** in your hospital?
   (1) Never (2) Sometimes (several times) (3) Usually
6. Do you think it is healthy about **your current diet habits**?
   (1) Very unhealthy (2) Little unhealthy (3) Moderate (4) Healthy (5) Very healthy
7. Overall, are you satisfied with **diet environment** of your hospital?
   (1) Very unsatisfied (2) Little unsatisfied (3) Moderate (4) Not bad (5) Very satisfied
8. In the aspect of diet environment, what do you want your **hospital to make more efforts in**? (You can choose multiple items)
   (1) None
   (2) Stop selling junk food in hospital
   (3) Promote healthy diet logo and classification of food in hospital
   (4) Include health diet requirement in the contract with catering manufacturers
   (5) Placing logos of calories and nutrition on diet sold in hospitals
   (6) Provide more healthy food or diet
   (7) Provide more nutrition promotion activities and education
   (8) Provide discount or incentives for staff to buy healthy diet
   (9) Promote counseling to catering manufacturers surrounding hospital
   (10) Provide nutrition certification to catering manufacturers surrounding hospital
   (11) Others: for example ______________________

■ **Stress coping**

1. Has your hospital publicized any stress coping information to the hospital staff?
   (1) Never (2) Little (3) Some (4) Many (5) A lot of
2. Have you ever attended **lectures** or educational activities related to **stress coping** in hospital?
   (1) Never (2) Sometimes (several times) (3) Usually

3. Have you ever done any screening for **stress level** in hospital?
   (1) No (2) Yes
4. Does the hospital offer **individual mental consulting services to hospital** staff?
   (1) No (2) Yes
5. Are there **leisure facilities** for hospital staff to use in hospital? (eg. garden path, reading room, music, coffee café, Wii, etc)
   (1) None or few (2) Not much (3) Some (4) Many (5) A lot of
6. Are there **extracurricular activities or clubs** in hospital?
   (1) None or few (2) Not much (3) Some (4) Many (5) A lot of
7. Have you ever attended **activities of these clubs**?
   (1) Never (2) Less than 1 time/ month (3) More than 1 time/ month
   (4) 1-2 times/ week (5) More than 3 times/ week
8. Are there **relaxing or stress-relieving facilities** designed especially for hospital staff to use in your hospital? (eg. meditation, sandbags outlet, etc)
   (1) No (2) Yes
9. Have you **ever used relaxing or stress-relieving facilities** in hospital?
   (1) Never (2) Less than 1 time/ month (3) More than 1 time/ month
   (4) 1-2 times/ week (5) More than 3 times/ week
10. Do you have opportunities to join any social clubs with other hospital staff in your hospital?
    (1) None or few (2) Not much (3) Some (4) Many (5) A lot of
11. What do you think about your current **stress coping** status?
    (1) Very bad (2) Not good (3) Not bad (4) Good (5) Very good
12. What do you think about **cross-sectorial interaction and collaboration** in hospital?
    (1) Very bad (2) Not good (3) Not bad (4) Good (5) Very good
13. Do you think that hospital pays attention to hospital staff's **opinion and participation**?
    (1) Little (2) Not much (3) Moderate (4) Much (5) Very much
14. Do you think that hospital regards "**staff"** as an important **resource** of hospital management and development?
    (1) Little (2) Not much (3) Moderate (4) Much (5) Very much
15. Do you think current resources of hospital **are helpful** for working staff to cope with stress?
    (1) Very unhelpful (2) Little helpful (3) Not bad (4) Helpful (5) Very helpful
16. Overall, are you satisfied with **the caring environment** in hospital?
    (1) Very unsatisfied (2) Little unsatisfied (3) Moderate (4) Not bad (5) Very satisfied

17. In which aspects of health promotion, you would like the hospital to make more efforts? (You can choose multiple items)
    (1) None
    (2) Providing more education related to stress coping
    (3) Providing more opportunities for stress screening
    (4) Provide more individual consulting services
    (5) Enhance relaxing and leisure facilities
    (6) Promote social interaction between hospital staff
    (7) Promote communication between staff and supervisor
    (8) Promote cross-sectoral collaboration
    (9) Promote hospital staff's participation in decision making process
    (10) Provide discount or financial incentives for hospital staff to attend social clubs or extracurricular activities
    (11) Others: for example ______________________

■ **Comprehensive opinion**

1. What kind of medical services do you think your hospital put more emphasis on, technical skills or whole person caring?
    (1) Technical skills >> Caring
    (2) Technical skills > Caring
    (3) Technical skills = Caring
    (4) Caring > Technical skills
    (5) Caring >> Technical skills
2. Do you feel that a good hospital staff needs to pay "Attention on **health promotion"**?
    (1) No (2) Little (3) Moderate (4) Agree (5) Totally agree
3. Do you agree that your hospital **has paid more attention on hospital staff's health** in recent years?
    (1) Very disagree (2) Disagree (3) Moderate (4) Agree (5) Very agree
4. Do you agree that **overall environment** of your hospital **becomes healthier** than before?
    (1) Very disagree (2) Disagree (3) Moderate (4) Agree (5) Very agree
5. Will increasing hospital's attention on staff's health and welfare increase your willingness to work retention?
    (1) Negative effect (2) No effect (3) Increase little (4) Increase some (5) Increase a lot
6. Overall, are you satisfied with your **working environment** in hospital?
    (1) Very unsatisfied (2) Little unsatisfied (3) Moderate (4) Not bad (5) Very

satisfied

7. Do you **care** about **future development** of this hospital?
   (1) Don’t care (2) Little care (3) Moderate (4) Care (5) Very care
8. Are you **proud of** being a member of this hospital?
   (1) No (2) Little (3) Some (4) Proud (5) Very proud

■ **Personal data**

1. What’s your **professional background**?
   (1) Physician (2) Nurse (3) Pharmacist (4) Other member with clinical medical license (5) Administrative, clerical, information, medical management, research, planning, etc work staff (6) Mechanic, worker, etc laborer (7) Others: _____________(Please explain)
2. Are you a **supervisor**? (1) Yes (2) No
3. Your **workplace** (You can choose multiple items):
   (1) Surgery or delivery room (2) Ward (3) Laboratory (4) Emergency or ICU (5) Outpatient (6) Administration sector (7) Others: _____________(Please explain)
4. Your **birth** date: Year of the Republic of China __________
5. What date do you start working **in this hospital**:
   Year of the Republic of China __________; Month ____________
6. **Gender**: (1) Male (2) Female
7. **Highest education degree**:
   (1) Below junior high school (2) Graduate from senior high school or higher vocational education (3) Graduate from specialist (4) Graduate from university (5) Graduate from graduate school
8. **Current marital status**:
   (1) Unmarried (2) Married (3) Married and separated (4) Divorced (5) Widowed
